# SARS-CoV-2 Exposure and Infection Among Health Care Personnel — Minnesota, March 6–July 11, 2020

**DOI:** 10.15585/mmwr.mm6943a5

**Published:** 2020-10-30

**Authors:** Ashley Fell, Amanda Beaudoin, Paige D’Heilly, Erica Mumm, Cory Cole, Laura Tourdot, Abbey Ruhland, Carrie Klumb, Josh Rounds, Brittney Bailey, Gina Liverseed, Molly Peterson, J.P. Mahoehney, Malia Ireland, Maria Bye, Sudha Setty, Maureen Leeds, Joanne Taylor, Stacy Holzbauer, Minnesota Department of Health COVID-19 HCW Monitoring Response Team, Minnesota Department of Health COVID-19 Response Task Force, Minnesota Department of Health COVID-19 HCW Monitoring Response Team, Nicky Anderson, Rich Austin, Chris Ballard, Nick Braun, Margaret Brown, Rachel Cahoon, Amy Casey-Paal, Dominique Cavallo, Ben Christianson, Pete Cole, Sarah Conboy, Jessica Davis, Karmen Dippmann, Karl Fernstrom, Lydia Fess, Barbara Frohnert, Patti Fuller, Ruth Furan, Paul Germann, Shelbi Giesel, Christopher Greene, Erin Hammell, Rachel Hannigan, Nicole Hedeen, Mike Hentges, David Hesse, Mona Highsmith, Catherine Johnson, Susan Kalis, Nathan Kemp, Nathan Kemp, Joan Kindt, Hannah Kinzer, Gretchen Koenigs, Alina Kraynak, Mary Beth Lacina, Susan LaMotte, Amy Longhenry, Jerry Malloy, Jamie Margetta, Olga Mastrodemos, Davis Melin, Laurie Menke, Michelle Messer, Stephanie Meyer, Greg Nelson, Jessica Nelson, James Noyola, Elizabeth Palmi, James Peacock, Tina Peters, Molly Peterson, Lauren Piper, Angela Serfling, Alyssa Steidl, Emily Styles, Erin Tibbetts, Marie Tran, Daniel Tranter, Brenda Waltz, Cynthia Wentkiewicz, Kari Witte, John Woodside, Minnesota Department of Health COVID-19 Response Task Force, Mary Ellen Bennett, Kathy Como-Sabetti, Richard Danila, Kris Ehresmann, Tammy Hale, Leslie Lovett, Ruth Lynfield, Karen Martin, Kirk Smith, Jacy Walters

**Affiliations:** ^1^Minnesota Department of Health; ^2^Council of State and Territorial Epidemiologists Fellowship Program, Atlanta, Georgia; ^3^Division of Healthcare Quality Promotion, National Center for Emerging and Zoonotic Infectious Diseases, CDC; ^4^Division of State and Local Readiness, Center for Preparedness and Response, CDC; ^5^Epidemic Intelligence Service, CDC.; Minnesota Department of Health; Minnesota Department of Health; Minnesota Department of Health; Minnesota Department of Health; Minnesota Department of Health; Minnesota Department of Health; Minnesota Department of Health; Minnesota Department of Health; Minnesota Department of Health; Minnesota Department of Health; Minnesota Department of Health; Minnesota Department of Health; Minnesota Department of Health; Minnesota Department of Health; Minnesota Department of Health; Minnesota Department of Health; Minnesota Department of Health; Minnesota Department of Health; Minnesota Department of Health; Minnesota Department of Health; Minnesota Department of Health; Minnesota Department of Health; Minnesota Department of Health; Minnesota Department of Health; Minnesota Department of Health; Minnesota Department of Health; Minnesota Department of Health; Minnesota Department of Health; Minnesota Department of Health; Minnesota Department of Health; Minnesota Department of Health; Minnesota Department of Health; Minnesota Department of Health; Minnesota Department of Health; Minnesota Department of Health; Minnesota Department of Health; Minnesota Department of Health; Minnesota Department of Health; Minnesota Department of Health; Minnesota Department of Health; Minnesota Department of Health; Minnesota Department of Health; Minnesota Department of Health; Minnesota Department of Health; Minnesota Department of Health; Minnesota Department of Health; Minnesota Department of Health; Minnesota Department of Health; Minnesota Department of Health; Minnesota Department of Health; Minnesota Department of Health; Minnesota Department of Health; Minnesota Department of Health; Minnesota Department of Health; Minnesota Department of Health; Minnesota Department of Health; Minnesota Department of Health; Minnesota Department of Health; Minnesota Department of Health; Minnesota Department of Health; Minnesota Department of Health; Minnesota Department of Health; Minnesota Department of Health.; Minnesota Department of Health; Minnesota Department of Health; Minnesota Department of Health; Minnesota Department of Health; Minnesota Department of Health; Minnesota Department of Health; Minnesota Department of Health; Minnesota Department of Health; Minnesota Department of Health; Minnesota Department of Health.

Health care personnel (HCP) are at increased risk for infection with SARS-CoV-2, the virus that causes coronavirus disease 2019 (COVID-19), as a result of their exposure to patients or community contacts with COVID-19 (*1*,*2*). Since the first confirmed case of COVID-19 in Minnesota was reported on March 6, 2020, the Minnesota Department of Health (MDH) has required health care facilities* to report HCP^†^ exposures to persons with confirmed COVID-19 for exposure risk assessment and to enroll HCP with higher-risk exposures into quarantine and symptom monitoring. During March 6–July 11, MDH and 1,217 partnering health care facilities assessed 21,406 HCP exposures; among these, 5,374 (25%) were classified as higher-risk^§^ (*3*). Higher-risk exposures involved direct patient care (66%) and nonpatient care interactions (e.g., with coworkers and social and household contacts) (34%). Within 14 days following a higher-risk exposure, nearly one third (31%) of HCP who were enrolled in monitoring reported COVID-19–like symptoms,^¶^ and more than one half (52%) of enrolled HCP with symptoms received positive SARS-CoV-2 test results. Among all HCP with higher-risk exposures, irrespective of monitoring enrollment, 7% received positive SARS-CoV-2 test results. Compared with HCP with higher-risk exposures working in acute care settings, those working in congregate living or long-term care settings more often returned to work (57%), worked while symptomatic (5%), and received a positive test result (10%) during 14-day postexposure monitoring than did HCP working outside of such settings. These data highlight the need for awareness of nonpatient care SARS-CoV-2 exposure risks and for targeted interventions to protect HCP, in addition to residents, in congregate living and long-term care settings. To minimize exposure risk among HCP, health care facilities need improved infection prevention and control, consistent personal protective equipment (PPE) availability and use, flexible sick leave, and SARS-CoV-2 testing access. All health care organizations and HCP should be aware of potential exposure risk from coworkers, household members, and social contacts. 

After detection of the first confirmed COVID-19 case in Minnesota on March 6, MDH requested that health care facilities provide a list of HCP who were exposed to persons with laboratory-confirmed SARS-CoV-2 infection. Health care facilities were asked to assess each exposure for evidence of higher risk for transmission. Higher-risk exposures occurred when HCP had close, prolonged contact with a person with confirmed COVID-19 or their secretions or excretions, while not wearing recommended PPE, or close, prolonged contact with persons with COVID-19 in the household or community (*3*). MDH staff members conducted 20-minute telephone risk-assessment interviews with HCP who had unknown or higher-risk exposure. The interviews included questions addressing how the exposure occurred, what type of PPE (if any) was worn, and whether a PPE breach occurred; MDH staff members made recommendations regarding quarantine and symptom monitoring. HCP who did not experience higher-risk exposure were asked to self-monitor for COVID-19–compatible signs or symptoms. For those HCP with higher-risk exposures, MDH recommended voluntary quarantine, including exclusion from work and community activities and daily MDH-supervised health monitoring via an emailed REDCap survey (*4*) for 14 days after the last known exposure. If COVID-19–like symptoms were experienced, HCP were encouraged to get tested. If the facility was experiencing a critical staffing shortage, HCP were told that they could return to work during their quarantine period if they were asymptomatic and wore appropriate PPE. HCP named during MDH interviews as close contacts of persons with confirmed COVID-19 were also assessed for exposure and enrolled into symptom monitoring. Information about exposed HCP was cross-referenced with MDH data on confirmed COVID-19 cases to identify HCP who potentially worked while infectious or received positive test results during monitoring. Descriptive analyses of HCP exposures and subsequent infection by exposure and facility type were conducted; facility types were categorized as acute care, ambulatory care, congregate living or long-term care, or other settings. A chi-squared test was used to assess the relationship between facility type and HCP exposure characteristics. This activity was reviewed by CDC and was conducted consistent with applicable federal law and CDC policy.**

During March 6–July 11, 2020, MDH and 1,217 participating health care facilities throughout Minnesota assessed 17,330 HCP for 21,406 exposures to a confirmed COVID-19 case in acute or ambulatory care patients (21% of exposures); residents in congregate living or long-term care settings (24%); coworkers (25%); congregate setting outbreak exposures (25%)^††^; and household or social contacts (5%). Among these, 5,374 (25%) were considered higher-risk exposures, 597 (11%) of which involved HCP caring for patients or residents at multiple facilities.

Among 4,020 (75%) HCP with higher-risk exposures for whom data were available, mean age was 39 years (range = 16–80 years). For 4,669 (87%) higher-risk exposures with available data, common HCP roles included nursing assistant or patient care aide (1,857; 40%), nursing staff members (1,416; 30%), administration (247; 5%), medical provider^§§^ (220; 5%), and environmental services (155; 3%).

Among 5,374 higher-risk exposures, 4,328 (81%) occurred in a health care setting, and 1,046 (19%) were related to household or social contacts. Among 4,328 exposures in health care settings, 1,380 (32%) involved patients in acute or ambulatory care, 1,185 (27%) involved congregate living or long-term care facility residents, 980 (23%) involved multiple infected HCP or residents in a congregate living or long-term care facility with four or more simultaneous cases, and 783 (18%) involved a coworker (Table 1). Higher-risk exposures involved direct patient care (66%) and nonpatient care interactions (e.g., with coworkers and social and household contacts) (34%). Among COVID-19 investigations that resulted in identification of one or more higher-risk HCP exposure, a single COVID-19 case in congregate living or long-term care resulted in higher-risk exposure of a median of three HCPs (interquartile range [IQR] = 1–6), compared with a median of one exposed HCP per case in acute or ambulatory care (IQR = 1–4). Across settings, an HCP with COVID-19 exposed a median of two coworkers (IQR = 1–3). Within 14 days following a higher-risk exposure, 373 (6.9%) of 5,374 HCP received a positive SARS-CoV-2 test result; HCP exposed to household or social contacts with COVID-19 had the highest positivity rate (13%) among all exposure types.

**TABLE 1:** Characteristics of higher-risk* SARS-CoV-2 exposures among health care personnel (HCP), by type of exposure investigation — Minnesota, March 6–July 11, 2020^†^

Characteristic	Exposures among health care personnel by type of exposure					
	Patient	Resident of congregate setting	Congregate setting outbreak exposures^§^	HCP	Household/Social	Total
No. (%) of higher-risk HCP exposures^§^	1,380 (26)	1,185 (22)	980 (18)	783 (15)	1,046 (19)	**5,374 (100)**
No. of confirmed COVID-19 investigations resulting in ≥1 higher-risk HCP exposure	305	280	100	259	919	**1,776**
Median (IQR) higher-risk HCP exposures per case	1 (1–4)	3 (1–6)	4 (1.5–12.5)	2 (1–3)	1 (1–1)	**1 (1–2)**
HCP with higher-risk exposures who received a positive SARS-CoV-2 test result within 14 days of exposure (%)	18 (1.3)	87 (7.3)	107 (10.9)	30 (3.8)	131 (12.5)	**373 (6.9)**
No. (%) of HCP enrolled in MDH monitoring	881 (63.8)	860 (72.6)	757 (77.2)	465 (59.4)	617 (59.0)	**3,580 (66.6)**
No. (%) of enrolled HCP who completed MDH monitoring	861 (97.7)	815 (94.8)	713 (94.2)	458 (98.5)	552 (89.5)	**3,399 (94.9)**
No. (%) HCP reporting symptoms during monitoring	250 (29.0)	302 (37.1)	224 (31.4)	143 (31.2)	141 (25.5)	**1,060 (31.2)**
Median (IQR) days from last exposure to symptom onset	8 (6–11)	7 (5–10)	7 (5–9)	8 (5–11)	4.5 (2–8)	**7 (5–10)**
No. (%) HCP tested for SARS-CoV-2 during monitoring	199 (23.1)	233 (28.6)	270 (37.9)	126 (27.5)	193 (35.0)	**1,021 (30.0)**
No. (%) HCP reporting symptoms who were tested for SARS-CoV-2 during monitoring	125 (50.0)	148 (49.0)	129 (57.6)	68 (47.6)	77 (54.6)	**547 (51.6)**

**Abbreviations:** COVID-19 = coronavirus disease 2019; IQR = interquartile range; MDH = Minnesota Department of Health.

* High-risk exposure category focused on eyes, nose, and mouth as primary routes of transmission, and HCP in this category lack protection of those areas. Medium-risk exposure category included potential for transmission by contamination of HCP hands or body during patient care activities. On May 19, CDC’s risk assessment was updated to include a single higher-risk exposure category. For this analysis, medium-, high- and higher-risk exposures were combined as “higher-risk exposures.” Higher-risk exposures assessed by MDH included nonpatient care interactions (e.g., coworkers, social contacts, and from household contacts).

^†^ The denominator includes all higher-risk HCP exposures, not the number of individual HCP exposed.

^§^ Outbreak exposures as defined by MDH involved four or more simultaneous COVID-19 cases in HCP or residents in the same congregate setting (i.e., HCP had potential for exposure to multiple positive individuals at time of exposure risk assessment).

Data on PPE use for acute care and ambulatory patient exposures were available for 913 higher-risk exposures; among these 822 (90%) HCP were wearing a medical-grade face mask or respirator and 240 (26%) were wearing eye protection. In comparison, when exposed to a congregate living or long-term care resident with COVID-19, significantly fewer HCP were wearing a medical-grade mask or respirator (611 of 905; 68%) or eye protection (140 of 905; 16%) (Table 2).

**TABLE 2:** Comparison of personal protective equipment (PPE) use and patient source control reported by health care personnel (HCP) during higher-risk* SARS-CoV-2 exposures in acute- and ambulatory-care settings and congregate living and long-term-care settings — Minnesota, March 6–July 11, 2020^†^

PPE and exposure characteristic	No. (%)		p-value^§^
	Acute- and ambulatory-care setting exposures (n = 913)	Congregate living/long-term care setting exposures (n = 905)	
HCP mask	757 (82.9)	565 (62.4)	<0.001
HCP respirator	65 (7.1)	46 (5.1)	0.07
HCP eye protection	240 (26.3)	140 (15.5)	<0.001
Patient/Resident mask	119 (13.0)	59 (6.5)	<0.001
HCP no PPE	92 (10.1)	131 (14.5)	0.004
HCP PPE breach	142 (15.6)	101 (11.2)	0.006
HCP in the same room when an AGP^¶^ was performed	167 (18.3)	34 (3.8)	<0.001

**Abbreviation:** AGP = aerosol-generating procedure.

* High-risk exposure category focused on eyes, nose, and mouth as primary routes of transmission and HCP in this category lack protection of those areas. Medium-risk exposure category included potential for transmission by contamination of HCP hands or body during patient care activities. On May 19, CDC’s risk assessment was updated to include a single higher-risk exposure category. For this analysis, medium-, high- and higher-risk exposures were combined as “higher-risk exposures.” Higher-risk exposures assessed by Minnesota Department of Health included nonpatient care interactions (e.g., coworkers, social contacts, and from household contacts).

^†^ The denominator includes all higher-risk HCP exposures, not the number of individual HCP exposed.

^§^ Calculated with chi-squared test.

^¶^ AGP included open suctioning of airway secretions, sputum induction, cardiopulmonary resuscitation, endotracheal intubation and extubation, noninvasive positive pressure ventilation (e.g., bilevel positive airway pressure and continuous positive airway pressure), bronchoscopy, manual ventilation; nebulizer was considered an AGP through March 26, 2020.

As of July 11, 3,580 (67%) HCP with higher-risk exposure had enrolled in MDH daily monitoring (Table 1) (Table 3). Among 3,399 HCP who completed 14-day monitoring, 1,060 (31%) reported COVID-19–compatible signs or symptoms during the monitoring period. Median interval between exposure and symptom onset was 7 days (IQR = 5–10 days). HCP working in group homes^¶¶^ had the highest test positivity rate (16%) during monitoring. HCP working in congregate living or long-term care settings more often worked following higher-risk exposures (57% versus 37%, p<0.001), worked while symptomatic (4.8% versus 1.3%, p<0.001), and received a SARS-CoV-2 positive test result during monitoring (9.6% versus 3%, p<0.001) than did HCP working in acute care.

**TABLE 3:** Characteristics of health care personnel (HCP) with higher-risk exposure, by type of facility where exposure occurred — Minnesota, March 6–July 11, 2020*

Characteristic	No. (%)							
	Type of facility							
	Type of congregate living/long-term care facility			All congregate settings	Acute care	Ambulatory care	Other settings^†^	Overall
	Skilled nursing	Assisted living	Group home					
HCP with higher-risk exposures	1,396 (26)	799 (15)	381 (7)	2,576 (48)	1,953 (36)	306 (6)	539 (10)	**5,374 (100)**
Facilities with confirmed COVID-19 investigations resulting in ≥1 higher-risk HCP exposure	113 (14)	165 (20)	145 (18)	423 (51)	78 (9)	127 (15)	199 (24)	**827 (100)**
Median (IQR) exposures per facility	5 (1–16)	2 (1–4)	1 (1–3)	2 (1–5)	5 (2–18)	1 (1–2)	1 (1–2)	**2 (1–4)**
HCP with higher-risk exposures who tested positive for SARS-CoV-2 within 14 days of exposure (% of all higher-risk exposures)	120 (8.6)	65 (8.1)	62 (16.3)	247 (9.6)	58 (3.0)	20 (6.5)	48 (8.9)	**373 (6.9)**
HCP enrolled in MDH monitoring	1,001 (28)	562 (16)	217 (6)	1,780 (50)	1,256 (35)	201 (6)	343 (10)	**3,580 (100)**
Enrolled HCP who completed MDH monitoring	951 (95.0)	520 (92.5)	197 (90.8)	1,668 (93.7)	1,217 (96.9)	196 (97.5)	318 (92.7)	**3,399 (94.9)**
HCPs reporting symptoms during monitoring	328 (34.5)	168 (32.3)	69 (35.0)	565 (33.9)	339 (27.9)	62 (31.6)	94 (29.6)	**1,060 (31.2)**
Median (IQR) days from last exposure to symptom onset	7 (5–10)	8 (6–9)	7 (4–10)	7 (5–10)	8 (5–10)	8 (6–11)	6 (3–10)	**7 (5–10)**
HCP tested for SARS-CoV-2 during monitoring	258 (27.1)	199 (38.3)	86 (43.7)	543 (32.6)	312 (25.6)	52 (26.5)	114 (35.8)	**1,021 (30.0)**
HCP asked to return to work^§^ during monitoring	591 (65.5)	316 (67.1)	115 (60.2)	1,022 (65.3)	463 (45.0)	78 (53.4)	158 (51.8)	**1,721 (56.6)**
HCP returned to work^§^ during monitoring	500 (55.4)	283 (60.1)	100 (52.4)	883 (56.5)	382 (37.2)	65 (44.5)	134 (43.9)	**1,464 (48.1)**
HCP reporting working with symptoms^§,¶^ during monitoring	41 (4.5)	25 (5.3)	9 (4.7)	75 (4.8)	13 (1.3)	3 (2.1)	7 (2.3)	**98 (3.2)**

**Abbreviations:** COVID-19 = coronavirus disease 2019; IQR = interquartile range; MDH = Minnesota Department of Health.

* The denominator includes all higher-risk HCP exposures, not the number of individual HCP exposed.

^†^ Other settings include home health, hospice, adult foster care, pharmacies, and mental health and substance use treatment centers.

^§^ The denominator for these results includes HCP that were enrolled in symptom monitoring after April 7, the date that MDH began asking HCP if they had been asked to return to work and if they had returned in daily symptom monitoring surveys. Denominators include acute-care hospital (n = 1,028), skilled nursing (n = 902), assisted-living facility (n = 471), group home (n = 191), outpatient (n = 146), other (n = 305), and total (n = 3,043).

^¶^ https://www.cdc.gov/coronavirus/2019-ncov/symptoms-testing/symptoms.html.

## Discussion

HCP working in congregate living or long-term care settings, including skilled nursing, assisted living, and group home facilities, were less likely to wear appropriate PPE; worked more often when they were symptomatic; and were more likely to receive a positive SARS-CoV-2 test result within 14 days of a higher-risk exposure than were HCP working in acute care settings. These data also highlight the need for awareness of nonpatient care SARS-CoV-2 exposure risks and for targeted interventions to protect HCP, in addition to residents, in congregate living and long-term care settings. HCP are at high risk for SARS-CoV-2 infection and could introduce and spread COVID-19 in skilled nursing and assisted living facilities, where risk for spread among residents and coworkers is high (*1*,*5*). Congregate living or long-term care facilities often experience PPE shortages and chronic staffing shortages, and workers frequently lack formal training in infection prevention and control practices (*6*). HCP working in multiple long-term care facilities have been shown to be at higher risk for infection (*7*). The disparate exposure risks identified by MDH through HCP risk assessment and monitoring highlight a need for consistent PPE access and infection prevention and control training, especially in congregate living and long-term care settings. Recommended PPE use might reduce the number of HCP quarantined, ease staffing shortages, and prevent HCP infection (*8*). 

The importance of interventions to prevent SARS-CoV-2 exposures for HCP is highlighted by the finding that approximately one third of higher-risk exposures resulted from exposures to coworkers and to household or social contacts with COVID-19, and not through patient care. HCP exposed to household or social contacts with COVID-19 also had the highest positivity rate among all exposure types. HCP exposures to persons with COVID-19 in social or household settings had the highest test positivity rates during the 14-day monitoring period. Whereas most HCP recognize potential risks to residents or patients, interactions between coworkers in breakrooms, nursing stations, or other gathering areas might lead to higher-risk exposures, because HCP might not practice social distancing or use PPE when they are not working in patient care situations. Genetic sequencing of SARS-CoV-2 isolated from HCP working in Dutch health care facilities demonstrated multiple facility introductions by HCP (*9*). In Minnesota, some HCP working in skilled nursing facilities had genetically diverse SARS-CoV-2 virus strains indicating that they were infected through community or household exposures (*1*). Health care facilities and HCP should recognize the risk for infection from nonpatient care exposures that contribute to infections among HCP. Facilities need to clearly communicate these risks and promote preventative practices, as well as establish monitoring and exclusion protocols for recognized exposures (*10*). Such efforts could reduce risk for onward transmission in the health care setting.

The findings in this report are subject to at least three limitations. First, facilities conducted their own initial risk assessment of exposed HCP, so some HCP exposures initially classified as no- or low-risk might have been misclassified. Second, not all exposed HCP were assessed by this program because some facilities did not provide a list of exposed HCP to MDH for risk assessment follow-up, and MDH was unable to reach all exposed HCP referred by facilities or enroll all those with higher-risk exposures into monitoring. Finally, some infections of monitored HCP occurring within 14 days of a workplace exposure might have resulted from unrecognized community exposure.

Implementation of a statewide standardized system for HCP exposure reporting and risk assessment and partnership between MDH and occupational health departments at acute care and ambulatory facilities, and nursing directors at congregate living or long-term care facilities enabled and strengthened HCP monitoring. A state-level comprehensive approach to HCP monitoring has enabled MDH and partners to 1) implement a program to identify HCP exposures and provide quarantine guidance and state-supervised symptom monitoring, which might have decreased the risk for SARS-CoV-2 transmission in health care settings; 2) describe HCP exposures, including those potentially associated with infection; and 3) deliver information about risk, testing recommendations, PPE, quarantine, and symptom monitoring directly to HCP. Data from this surveillance program have highlighted the need for HCP to recognize the risk for SARS-CoV-2 exposure risks not associated with patient care and for targeted interventions to protect HCP, in addition to residents, in congregate living and long-term care settings.

**Summary**

**What is already known about this topic?**

Health care personnel (HCP) are at increased risk for COVID-19 from workplace exposures.

**What is added by this report?**

Among 21,406 Minnesota SARS-CoV-2 HCP exposures, 5,374 (25%) were higher-risk (within 6 feet, ≥15 minutes, or during an aerosol-generating procedure); exposures involved patient care (66%) and nonpatient contacts (34%). Compared with HCP working in acute care settings, those working in congregate living and long-term care more often worked while symptomatic and received positive SARS-CoV-2 test results.

**What are the implications for public health practice?**

HCP should recognize potential exposures unrelated to patient care and use prevention measures, including masks. HCP in congregate living and long-term care settings experience considerable risk and pose a transmission risk to residents. Improved access to personal protective equipment, flexible medical leave, and testing is needed.

## Footnotes

* Health care facilities as defined by MDH include acute care hospitals, critical access hospitals, long-term acute care hospitals, skilled nursing facilities, assisted living facilities, group homes, adult foster care, treatment facilities, dialysis centers, outpatient clinics, dental clinics, home health care, and hospice.

† HCP as defined by MDH include, but are not limited to, emergency medical service personnel, nurses, nursing assistants, physicians, technicians, therapists, phlebotomists, pharmacists, students and trainees, contractual staff members not employed by the health care facility, and persons not directly involved in patient care, but who could be exposed to infectious agents that can be transmitted in the health care setting (e.g., clerical, dietary, environmental services, laundry, security, engineering and facilities management, administrative, billing, and volunteer personnel). HCP does not include clinical laboratory personnel.

§ During February 8–May 18, 2020, CDC exposure risk assessment guidance included medium- and high-risk categories, with risk level based on PPE worn and type of potential contact with a person with confirmed COVID-19. On May 19, CDC’s risk assessment was updated to include a single higher-risk exposure category to include close (within 6 feet), prolonged (≥15 minutes or of any duration during an aerosol-generating procedure) contact with a person with confirmed COVID-19 or their secretions or excretions, while not wearing appropriate PPE (https://www.cdc.gov/coronavirus/2019-ncov/hcp/guidance-risk-assesment-hcp.html), or close, prolonged contact with cases in the household or community (https://www.cdc.gov/coronavirus/2019-ncov/hcp/guidance-risk-assesment-hcp.html). For the purpose of this analysis, medium-, high- and higher-risk exposures were combined into a single variable and identified as “higher-risk exposures.” Higher-risk exposures assessed by MDH included nonpatient care interactions (e.g., coworkers, social contacts, and from household contacts).

¶ https://www.cdc.gov/coronavirus/2019-ncov/symptoms-testing/symptoms.html.

** 45 C.F.R. part 46, 21 C.F.R. part 56; 42 U.S.C. **241(d); 5 U.S.C. **552a; 44 U.S.C. **3501 et seq.

†† Outbreak exposures as defined by MDH involved four or more simultaneous cases in HCP or residents in the same congregate setting (i.e., HCP had potential for exposure to multiple person with COVID-19 at time of exposure risk assessment).

§§ The medical provider category as defined by MDH includes medical doctor, doctor of osteopathy, nurse practitioner, and physician assistant.

¶¶ Group homes as defined by MDH included licensed home and community-based services, intermediate care for persons with intellectual disabilities, child foster Services, supervised living facilities, and boarding care.
